# Impact of image segmentation on high-content screening data quality for SK-BR-3 cells

**DOI:** 10.1186/1471-2105-8-340

**Published:** 2007-09-14

**Authors:** Andrew A Hill, Peter LaPan, Yizheng Li, Steve Haney

**Affiliations:** 1 Department of Biological Technologies, Wyeth Research, 35 CambridgePark Drive, Cambridge, MA 02140, USA

## Abstract

**Background:**

High content screening (HCS) is a powerful method for the exploration of cellular signalling and morphology that is rapidly being adopted in cancer research. HCS uses automated microscopy to collect images of cultured cells. The images are subjected to segmentation algorithms to identify cellular structures and quantitate their morphology, for hundreds to millions of individual cells. However, image analysis may be imperfect, especially for "HCS-unfriendly" cell lines whose morphology is not well handled by current image segmentation algorithms. We asked if segmentation errors were common for a clinically relevant cell line, if such errors had measurable effects on the data, and if HCS data could be improved by automated identification of well-segmented cells.

**Results:**

Cases of poor cell body segmentation occurred frequently for the SK-BR-3 cell line. We trained classifiers to identify SK-BR-3 cells that were well segmented. On an independent test set created by human review of cell images, our optimal support-vector machine classifier identified well-segmented cells with 81% accuracy. The dose responses of morphological features were measurably different in well- and poorly-segmented populations. Elimination of the poorly-segmented cell population increased the purity of DNA content distributions, while appropriately retaining biological heterogeneity, and simultaneously increasing our ability to resolve specific morphological changes in perturbed cells.

**Conclusion:**

Image segmentation has a measurable impact on HCS data. The application of a multivariate shape-based filter to identify well-segmented cells improved HCS data quality for an HCS-unfriendly cell line, and could be a valuable post-processing step for some HCS datasets.

## Background

Anticancer drug development is a highly complex process that explicitly models cancer cell growth in the laboratory. These cell models, usually tumor cell lines adapted to culture *in vitro *from human tumor samples, are chosen for use in pathway and target based research because of particular properties these lines retain, including the characteristics of the tissue of tumor origin, hormone responsiveness and genetic alterations that result in specific pathways becoming constitutively activated [1]. As such, certain cell lines are used in specific drug development programs because they appropriately model specific aspects of cellular signalling or tumor biology. One example is the breast carcinoma line SK-BR-3, in which the PI3K pathway is constitutively activated by both EGFR and the related Her-2^neu ^receptor [2-4]. This cell line is valuable to the study of inhibitors of the EGFR receptor family and the PI3K/AKT pathway.

High content screening (HCS) refers to the image-based analysis of cellular morphology [5]. In a typical experiment, monolayer cell cultures are fixed, stained with organelle- or cellular-component-specific fluorescent markers, and then imaged by automated microscopy. Images are "segmented" to identify cells or sub-cellular structures, and morphological "features" (such as fluorescent intensity, object shape, size and texture) are computed from each segmented object. The term "high content" refers to the very large volume (and potentially rich) datasets that can be generated by this approach. For example, an experimental protocol with 4 distinct fluorescent markers that computes 50 features per fluorescent marker will produce 200 feature measurements per cell, for each of ~10^5 ^cells in a single culture plate, yielding ~2 × 10^7 ^data points. HCS is gaining rapid acceptance as a methodology for quantitating cellular morphology *in vitro *[6-10].

The application of HCS to some important cell types can be restricted by limitations in the segmentation step of the analysis. In this step, microscopic images are processed by segmentation algorithms to locate and define cells or sub-cellular structures in a background of instrumentation noise and any non-cellular objects (debris, artefacts, etc.) that may appear in an image [e.g. [11]]. Segmentation algorithms work best on cell types where individual cells are uniform in size and shape, and grow in a regular non-overlapping pattern, because such cells are easier for the algorithms to distinguish from non-cell background. However, many clinically relevant human tumor cell lines such as SK-BR-3 grow in more complex patterns. For these "HCS unfriendly" cell lines, image segmentation is less successful, and errors in segmentation can occur frequently. For example, neighbouring cells may be inappropriately identified as a single object, or a cell body may be "over-segmented" or fragmented into several distinct objects. Errors in segmentation cause multiple or partial cells to be inappropriately designated as single cells, and can therefore distort downstream analyses of cellular features that are derived from the segmented objects. This is especially true in the context of a typical HCS screen, where hundreds or thousands of images are segmented, and it is not feasible for investigators to visually review all the acquired images.

Novel cell segmentation algorithms are under constant development, and have the potential to reduce segmentation errors. However, commercial HCS systems that are in use today typically use a repertoire of well-understood segmentation algorithms. This makes commercial systems powerful and easy to use, but limits the ability of users to incorporate novel image segmentation methods into their analysis processes.

To investigate segmentation issues in the context of commercial HCS informatics systems, we sought to determine if segmentation errors were common for the clinically relevant cell line SK-BR-3, if such errors had a measurable effect on the data, and if we could improve data quality by identifying and removing poorly segmented objects from datasets generated by a commercial HCS system.

## Results

### Poorly segmented objects are identified by human review of images

To provide a reference set of well- and poorly-segmented cells, we undertook human review of composite cell images from a selection of wells treated with CCI-779, HKI-272, PP3, SB 203580, Trichostatin A, or DMSO vehicle. For each image, we recorded cells that were well-segmented, and those that were poorly-segmented. Criteria for "good" segmentation were:

1. The cell perimeter defined in channel 1 was complete and reasonably conformed to the perimeter observed by eye in channel 1.

2. The nuclear perimeter defined in channel 2 was complete and enclosed in the surrounding channel 1 cell body.

**Figure 1:**
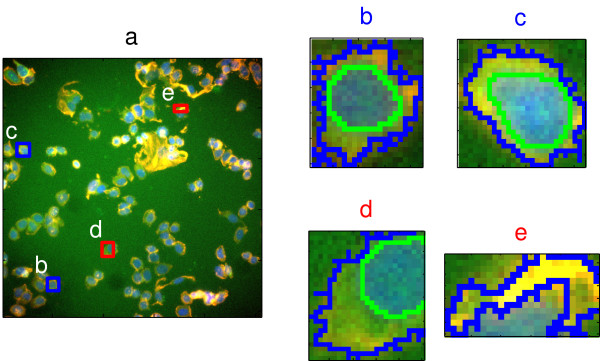
Image segmentation is imperfect. (a) Representative composite image from the training image set. Bounding boxes determined from cell-body stain are indicated for segmented objects that were classed by human review as either poorly segmented (red boxes) or well segmented (blue boxes). Letters (b-e) indicate individual segmented objects. Nuclear stain (DAPI) is blue; Cell body (CMFDA) is green; Actin is red. (b-c) Magnified images of well-segmented objects containing a complete nucleus and cytoplasmic region. Green outlines indicate segmented nuclei; blue outlines delimit the segmented cell body. (d-e) Magnified images of poorly-segmented objects containing partial cell bodies and nuclei.

Each of the four authors of this report independently reviewed about one-quarter of the total images. Typical examples of well- and poorly-segmented objects are shown in Figure 1. The well segmented objects tended to be circular or ellipsoidal in shape, with regular perimeters (Figure 1b, 1c), while the poorly segmented objects tended to be irregular shaped partial cell bodies (Figure 1d), often with no identified nuclear region (Figure 1e).

In total, 2019 segmented cells were classified by human review: 719/2019 were considered well-segmented, and 1300/2019 poorly-segmented. The fact that 64% of the segmented objects were classed as poorly-segmented was striking, and suggested that segmentation errors might be introducing substantial noise and/or bias into our HCS data for this cell line. Further evaluation of other SK-BR-3 cultures confirmed that approximately one-half of segmented objects were of poor quality, so this finding was not atypical.

The set of 2019 human-reviewed segmented cells was split by a one-time random division into a training set of 1009 training cases and 1010 test cases. The feature data calculated by the Cellomics software for these segmented objects was then used for training and validation of classifiers to distinguish well- and poorly-segmented cells.

### Training of classifiers to distinguish poorly-segmented cells

**Figure 2:**
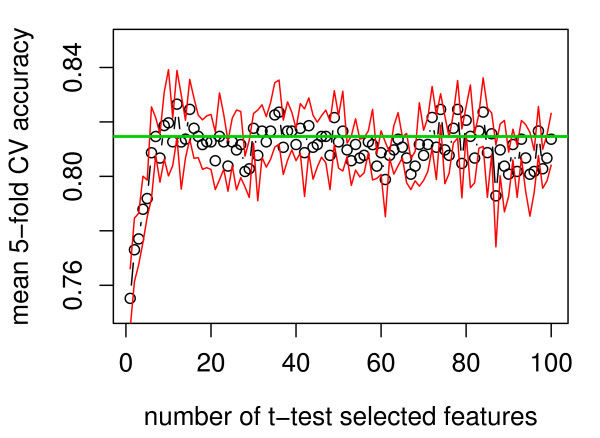
Cross validation indicates a small number of morphological shape features are sufficient to distinguish well- and poorly-segmented objects. Five-fold cross validation was executed on the training set, including feature selection in each round of training. Fraction of correctly classified training cases (from a total of 1009) is shown as a function of the number of morphological features in the SVM-RBF classifier. The open circles indicate mean accuracy; the red lines delimit one standard error around the mean. The green horizontal line marks the accuracy for 7 features, which was the minimal number of features for which the accuracy was within one SEM of the maximum.

Using only the 1009 segmented cells in the training set, we built classifiers to identify poorly-segmented cells. The training data set consisted of the 116 image features that were computed by the Cellomics software for all of the 1009 training cells. We evaluated a linear discriminant (LDA) classifier, and two support-vector machine (SVM) classifiers, with either linear (SVM-linear), or radial-basis function (SVM-RBF) kernels. For each of these classifiers, we executed five-fold cross validation within the training set to assess classification success, for 1–100 classifying features, selected by a t-test comparing well- and poorly-segmented cells. For each classifier, the smallest number of features that gave a 5-fold cross-validation accuracy within one standard error of the maximum achieved accuracy was identified. Table 1 shows these "one-SEM" accuracies of all 3 classifiers in cross-validation, and the corresponding number of features. The accuracies of the LDA, SVM-linear, and SVM-RBF in this cross-validation were 79.5–81.5%, so all 3 models performed very similarly. Figure 2 shows the 5-fold cross validation accuracy of the SVM-RBF classifier, for 1 to 100 features. The 7-feature model performance was within one standard error of maximum. Performance in cross-validation reached a plateau for larger feature numbers. The gamma and cost parameters of the 7-feature SVM-RBF model were optimized by grid search and 10-fold cross validation in the training set (final parameter values were gamma = 0.333, cost = 1).

**Table 1:** Performance of classifiers on training and test sets

	**5-fold cross-validation**		**Independent test set**
		
**Classifier**	**Features**	**Accuracy**	**Accuracy**
LDA	19	79.5%	78.7%
SVM-linear	25	80.1%	78.9%
SVM-RBF	7	81.5%	80.9%

LDA, SVM (linear kernel), and SVM (radial basis kernel) classifiers were first assessed by 5-fold cross validation accuracy for a range of model sizes. The minimum number of features that gave a cross-validation accuracy within one SEM of the maximum accuracy for all model sizes was determined, and is shown in the first column of the table, along with the corresponding cross-validation accuracy. Once the optimal parsimonious model size (number of features) was determined from cross-validation, the classifiers were applied to the independent test set; the test-set accuracy is shown in the right-most column of the table.

### Testing of classifiers on independent test cells

When training of the classifiers was complete, we tested the final trained LDA, SVM-linear, and SVM-RBF models on our test set of 1010 segmented cells. These cells were not used for model training, and therefore represented an independent validation dataset for our models. Performance of the models on the test set was generally consistent with the cross-validation results. In particular the SVM-RBF classifier gave 80.9% accuracy (Table 1). The confusion matrix in the test set for the SVM-RBF classifier indicated that classification of poorly segmented objects was somewhat higher than that for well-segmented objects (Table 2). Since the SVM-RBF classifier gave the highest overall accuracy with the minimal number of features, we selected that classifier for further analysis.

**Table 2:** Test set confusion matrix for the SVM-RBF classifier

	**True Class**	
	
**Predicted Class**	PS	WS
PS	549	78
WS	115	268

Of 1009 test cases, 80.9% were correctly classified by the SVM-RBF classifier. The confusion matrix shows that accuracy of classification for poorly segmented (PS) objects was somewhat better than that for well-segmented (WS) objects.

### Several of the most powerful discriminant features are shape descriptors

**Figure 3:**
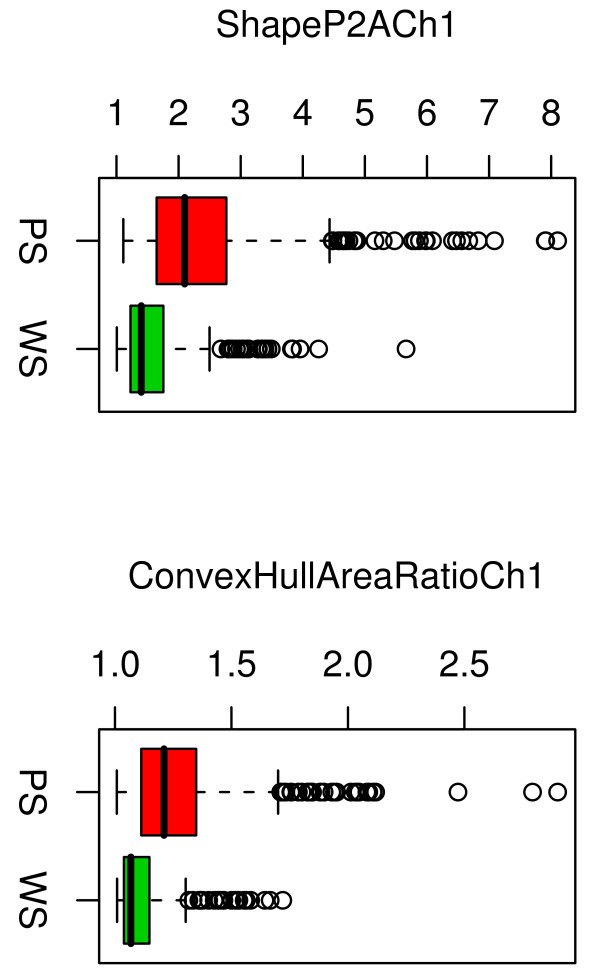
Shape parameters of poorly-segmented (PS) and well-segmented (WS) cells. Data for 50 training cells is shown. Well-segmented objects tended to be more circular and less variable in shape than poorly-segmented objects.

The 116 morphological features that we included in our analysis were generated by the Cellomics Morphology Explorer application and included measures of total intensity, average intensity, object size, object shape, and object texture. The seven features selected for the SVM-RBF classifier included 2 nuclear intensity measures (AvgIntenCh2, TotalIntenCh2), and one cell body texture measure (EntropyIntensityCh1). However, more than half (4 of 7) of the model features were cell-body shape measures (ConvexHullAreaRatioCh1, ConvexHullPerimRatioCh1, FiberWidthCh1, and ShapeP2ACh1). Review of the P2A and ConvexHullAreaRatio features for the training cells indicated that the well-segmented objects tended to have lower values than poorly-segmented objects for both these features (Figure 3). These lower values indicated that well-segmented objects tended to be more circular, with fewer bumps or protrusions, compared to the poorly-segmented objects. This was generally consistent with our visual observations of these objects (e.g. Figure 1).

Once we established that the classifier could mimic the human definition of well- and poorly-segmented objects, we applied it to experimental data to determine if identifying and removing poorly-segmented objects could improve the quality of our HCS readouts for SK-BR-3 cells. To assess data quality we focussed on two aspects of the data: DNA content distributions in control cells, and morphological feature readouts from control and perturbed cells.

### DNA content of well-segmented cells is free of debris signal and consistent with the expected log-phase distribution

**Figure 4:**
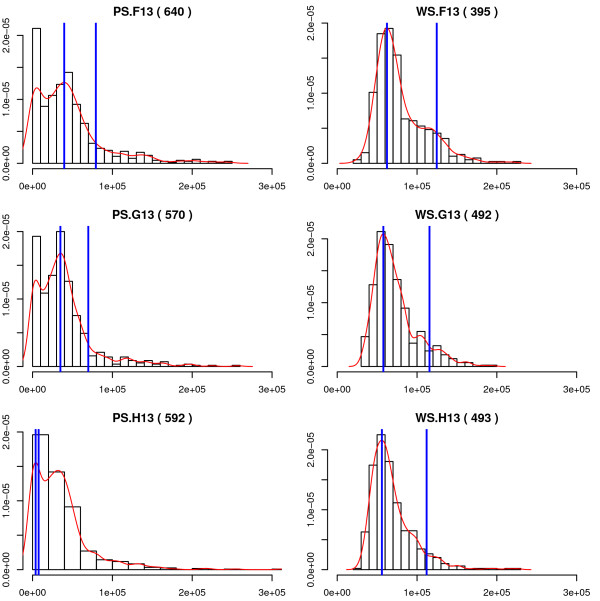
DNA content of poorly-segmented (PS) and well-segmented (WS) objects. Each plot is a histogram of total DNA content from a single representative DMSO vehicle-treated well (F13, G13, or H13). The left column shows DNA content of poorly-segmented objects; well-segmented objects from the same wells are shown in the right column. The number in parentheses above each plot indicates the total number of objects included in the histogram. The red curve is a smoothed fit to the observed distribution. The blue lines were placed at the mode of the fitted distribution (the presumptive G0/G1 peak), and at twice the mode (the expected location of the G2/M peak). Note the poor estimates of the location of the G0/G1 peak in the poorly-segmented-class histograms, due to the large debris peak at small DNA content.

Our vehicle-treated cultures were in log-phase growth. Therefore we expected the distribution of DNA content per cell to follow the classical bi-modal distribution with a major peak at 2N DNA content corresponding to G0/G1 cells, a minor peak at 4N containing G2/M phase cells, and subset of S-phase cells between 2N and 4N DNA content. We examined the histograms of total intensity in Channel 2, which measured DNA content, for objects classified as poorly-segmented, compared to that of those classed as well-segmented. The poorly-segmented objects showed a prominent "debris" peak at very low DNA content, and a broad distribution of higher DNA contents. In contrast, well-segmented objects had little or no debris population, a sharper G0/G1 peak, and reduced spread of large DNA content objects beyond the G2/M peak (Figure 4). We confirmed by visual inspection of images that the "debris" peak contained cell fragments or artefacts, the G0/G1 peak contained singlet cells, and the G2/M peak contained both G2 and M phase cells (data not shown).

### Morphological feature variability is maintained in the well-segmented population

If the well-segmented cell population is to be selected for experimental analysis, it is important that this population be a representative sample of the cells of biological interest, and not an inappropriately homogeneous subset of cells with atypical morphology. To assess the heterogeneity of well-segmented cells, we compared the variation in morphological feature values within the well-segmented and poorly-segmented populations of vehicle-treated control cells.

**Figure 5:**
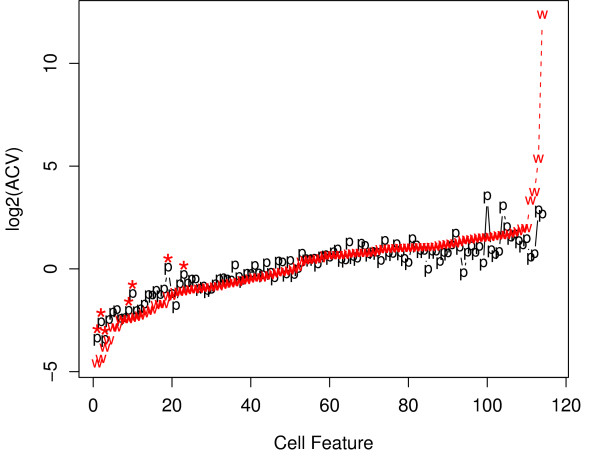
Variability in the well- and poorly-segmented populations. The absolute value of the coefficient of variation (ACV) was computed for each of the 116 morphological features, within well- and poorly-segmented populations from a representative set of DMSO-vehicle treated cultures. Each population contained approximately 3300–4000 cells. Features were sorted by their ACV in the well-segmented population for display purposes. The red line labelled "w" indicates ACV for features in the well-segmented population; the black line labelled "p" is ACV of the same features in the poorly segmented population. The asterisks indicate the 7 features that were used in the SVM-RBF classifier. The overall similarity in ACV between the two populations indicates that the well-segmented population retained significant biological variation of interest.

The absolute value of the coefficient of variation (ACV) of each of the 116 morphological features was calculated, for both well- and poorly-segmented populations. Data from 7 representative wells was used, so that each ACV was estimated from a reasonably large population of approximately 3300–4000 cells. The ACVs of each of the 7 features that were used to define the well-segmented population were reduced within that population, as expected (Figure 5). The median CV of all 116 cell features was 125% in the poorly-segmented population, and 142% in the well-segmented population. The number of features with smaller variation in the well-segmented population was 57 of 116 (49%). Hence, there appeared to be no inappropriate reduction in the overall heterogeneity of the well-segmented population, compared to the poorly-segmented population.

### Dimension and shape features of the cell body are most sensitive to poor segmentation

The features used by the classifier to define well- and poorly-segmented cells were intensity, cell body object shape, and texture features. We expected that other features would be more or less sensitive to poor cell segmentation, according to their individual characteristics. For example, we speculated that texture features might be less sensitive to cell body segmentation than object shape features. To test this, we fitted the dose responses of all features to the subset of compounds that showed significant dose responses by ANOVA, in at least some features. We carried out this fitting twice: first using only poorly-segmented objects as determined by our classifier, and then using only the well-segmented objects. We then calculated a measure of the error-weighted discrepancy between the poorly-segmented object dose response and the well-segmented dose response, and categorized each compound-feature combination, as either "sensitive" to segmentation (if the discrepancy was larger than the grand median) and "resistant" (if the discrepancy was smaller than the grand median).

**Figure 6:**
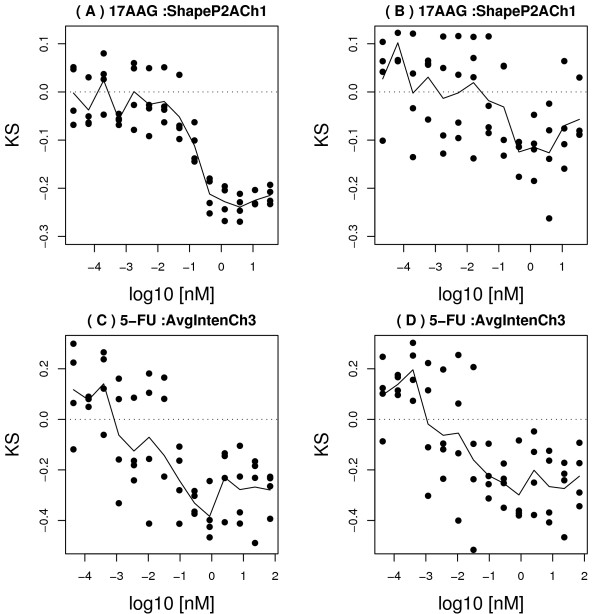
Examples of segmentation-sensitive and resistant cell-body features. Panels (A, B) show dose response of the KS statistic to the compound 17AAG, for the P2A feature in Channel 1, computed using only well-segmented cells (A), or only poorly segmented cells (B). A substantial difference is seen between the two responses for this "sensitive" feature. Panels (C, D) show dose response of KS statistic to the compound 5-FU, for the Average Intensity feature in Channel 3, computed using only well-segmented cells (C), or only poorly segmented cells (D). The two dose responses are similar for this "resistant" feature.

We found that there was a wide range of sensitivity to cell body segmentation among the compound-feature combinations. "Sensitive" compound-features showed very discrepant dose responses in well- versus poorly-segmented cells (e.g. Figure 6a,b) while "resistant" compound-features showed consistent responses regardless of segmentation status (e.g. Figure 6c,d).

To clarify the varying sensitivity of features to cell body segmentation, we tabulated the number of drug-feature combinations in each channel that were either sensitive or resistant to segmentation, and grouped features by their type, where type was either dimension, intensity, shape, or texture (Table 3). From this tabulation, two clear trends emerged.

**Table 3:** Sensitivity of compound-feature combinations to cell segmentation

		**Resistant**	**Sensitive**	**Overall Sensitive %**
		
**Channel**	**Feature Type**			
			
Ch1	arrangement	24	16	58%
	dimension	16	94	
	intensity	32	18	
	shape	33	27	
	texture	14	6	
Ch2	intensity	19	1	5%
Ch3	arrangement	6	14	47%
	intensity	36	14	
	texture	26	33	
Ch4	arrangement	10	20	46%
	dimension	7	3	
	intensity	57	33	
	shape	2	8	
	texture	32	28	

The dose response of each feature for each drug was fitted by a linear model, using either the well-segmented cell population, or the poorly-segmented cell population. The discrepancy between the well- and poorly- segmented dose responses was calculated for each drug-feature combination, and drug-feature combinations were then binned as either sensitive to segmentation (if the discrepancy was larger than the grand median discrepancy) or resistant to segmentation (if the discrepancy was smaller than the grand median).

First, the nuclear staining in Channel 2 tended to be more resistant to Ch1 segmentation quality than Channels 1, 3 or 4 (Table 3). The fraction of sensitive compound-feature combinations was 5% in Channel 2, versus 46–58% in Channels 1, 3 and 4. This was not surprising, since DAPI staining of nuclear regions in Channel 2 tended to be robust and largely independent of the segmentation of the cell body in Channel 1. Conversely, the size of the regions that were segmented in channels 3 and 4 depended strongly on the delineation of the cell body in Channel 1, and this dependence seems to be reflected in the sensitivity of Channel 3 and 4 features to cell body segmentation in Channel 1.

Secondly, within Channel 1, dimension and shape features tended to be more sensitive to cell segmentation than did intensity or texture features (Table 3). Specifically, 94/110 dimension features were sensitive and 27/60 shape features were sensitive, whereas only 18/50 intensity features and 6/20 texture features were sensitive. Since 4 of the 7 features in our SVM-RBF classifier were shape features (ConvexHullAreaRatio, ConvexHullPerimRatio, P2A, and FiberWidth in Ch 1), it makes intuitive sense that Ch1 shape features should be very dependent on segmentation class. Conversely, intensity and texture features, although they are calculated using the segmented regions from Channel 1, are not inherently dependent on cell shape. Therefore it appears sensible that they should be less sensitive to the quality of segmentation of the cell body in Channel 1.

### Observable morphological changes are captured by analysis of the well-segmented cell population, but not the poorly-segmented population

Given the sensitivity of some morphological feature measurements to segmentation (e.g. Figure 6a,b), we next asked if limiting analysis to well-segmented objects could enhance our ability to detect true morphological changes in the cell body of compound-treated cells.

**Figure 7:**
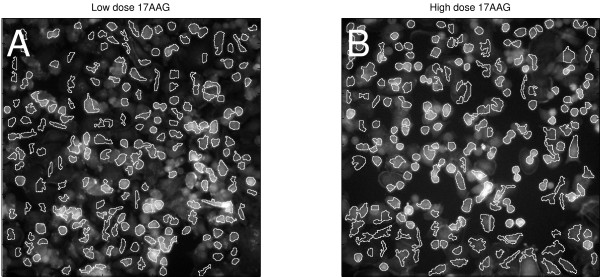
Effect of 17AAG on cell shape. Grayscale images in Channel 1 are shown. (A) Cells after exposure to low dose (0.02 pM) 17AAG. (B) After exposure to high dose (35 nM) 17AAG. White overlay shows the segmented cell body regions.

For the 17AAG-treated cells of Figure 6a–b, we reviewed the raw cell images in Channel 1, for low- and high-dose treated cells. Representative field images confirmed that cells treated with high-dose (35nM) 17AAG tended to be rounder than at low dose (0.02 pM; Figure 7a,b). This was consistent with the observed decrease in perimeter-to-area ratio in Channel 1 (Figure 6a), but this morphological change was not captured by the same feature among poorly-segmented cells (Figure 6b). Hence, an observable change in shape of cells at high-dose 17-AAG was captured by analysis of the well-segmented cell population, but not by the poorly-segmented population.

**Figure 8:**
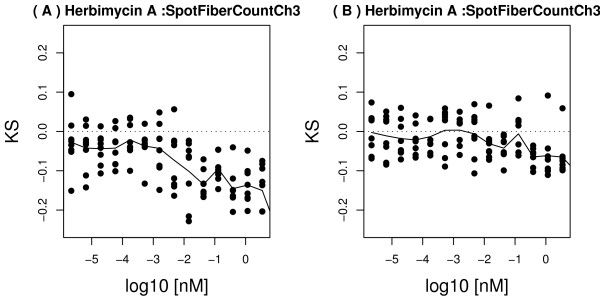
Examples of segmentation-sensitive and resistant actin-channel features. The dose response of the KS statistic to the compound Herbimycin A, for the FiberCount feature in Channel 3, computed using only well-segmented cells (A), or only poorly segmented cells (B).

**Figure 9:**
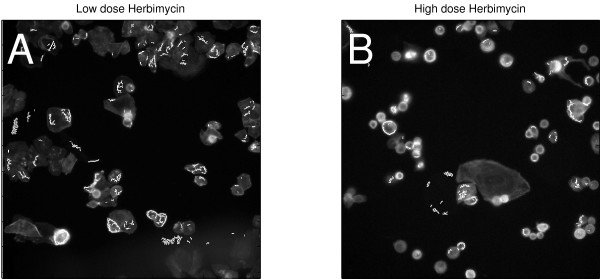
Effect of Herbimycin on actin fiber morphology. Grayscale images in Channel 3 are shown. (a) Cells after exposure to low dose Herbimycin. (b) After exposure to high dose Herbimycin. White overlays show the segmented actin fiber objects.

Next, we asked if morphological changes in a non-cell body channel were also better captured by analysis of the well-segmented cell population. We selected SpotFiberCountCh3, a count of the number of actin fibers in the cell body. This was a segmentation-sensitive feature that had a statistically significant dose-response to Herbimycin in the well-segmented cell population (p = 2.5 × 10^-9^), but an attenuated dose-response for the same treatment among the poorly-segmented population (Figure 8a,b). We reviewed representative field images for low- and high-dose treated cells, and found that actin fiber structures were more frequent in cells treated with low-dose Herbimycin, compared to high-dose Herbimycin (Figure 9a,b). This was consistent with the statistically significant downward trend in SpotFiberCh3 among the well-segmented cell population (Figure 8a), but this morphological change was not as strongly reflected in the poorly segmented population (Figure 8b).

## Discussion

Our review of segmented images of SK-BR-3 cells demonstrated that a large number (more than one-half) of the segmented objects in these images were of questionable quality. These objects were either partial cell bodies, or groups of multiple cells that had been inappropriately co-segmented. Our training cell images suggest some likely causes of this poor segmentation. First, SK-BR-3 cells tended to grow in closely spaced overlapping clusters of cells. This growth pattern makes segmentation intrinsically difficult, and tended to cause to under-segmentation (inappropriate merging of multiple cells). Second, it is possible that we had non-optimal image segmentation algorithm or parameter settings in our software. Non-optimal algorithms or parameters could explain over- or under-segmentation (inappropriate division of single cells into multiple objects). However, we used test images to optimize our segmentation parameters for our experiments, so our parameter settings should represent reasonable choices. Finally, variation in image quality in this large screening dataset may have adversely affected our image segmentation quality for some images. We did observe some images that were of variable intensity, or had poor focus. We expect that a combination of all three of these causes (cell growth properties, limitations of our segmentation algorithms, and variable image quality) contributed to some extent to poor segmentation in our dataset.

The segmentation challenges we observed are likely to be present in other HCS datasets, not just ours. For example, we expect that SK-BR-3 cells are not unique in their "HCS-unfriendly" morphology. Other clinically relevant cell lines also grow in similar patterns and therefore they may also be challenging for current segmentation algorithms. On the other hand, it is important to note that there are cell lines that grow in more regular patterns. For example, HeLa cells are regularly shaped and at appropriate densities can be relatively easy to segment. When we applied our shape-based classifier to HeLa cultures, the fraction of poorly segmented objects was much lower than in SK-BR-3 (approximately ~10% versus > 50%, data not shown). This demonstrates that the magnitude of the segmentation problem is very dependent on the cell type.

To address segmentation challenges in our SK-BR-3 cells, we developed a post-processing filtration step that incorporated a multivariate object shape classifier. An obvious alternative to our method of post-filtering of segmented objects would be to improve the image segmentation algorithm itself, so that more objects are correctly segmented to begin with. Such an approach is certainly logical and should be pursued. But in practice, we and many other HCS users use segmentation algorithms that are part of software packages with limited scope for modification. Hence, until routine segmentation can be made more robust for cell lines like SK-BR-3, our approach appears to have practical utility for users of commercial HCS systems.

Visual inspection of images from SK-BR-3 cultures suggested that well-segmented objects tended to have similar shapes and intensities. Cross-validated feature selection and our independent test set confirmed that 7 image features (2 intensity features, 4 cell body shape features, and 1 texture feature) optimally distinguished well- and poorly-segmented objects from one another in our SVM-RBF classifier. Of the features in our classifier, the shape parameters are most likely to have similar distributions in other cell types, and therefore generalize to the identification of well segmented cells in other cell lines. The applicability of our classifier to cell lines other than SK-BR-3 would need to be tested by re-evaluation of the filter on other cell types.

To further understand the impact of the shape-based filter on our data, we examined the sensitivity to segmentation of all our morphological features in each of our four channels, corresponding to cell body, nucleus, actin, and tubulin. We found that some features showed very different dose responses to compounds in the well-segmented cell population, compared to the poorly-segmented population, while others showed similar dose responses in either population. This was not surprising, since different types of features in different channels reflect very different measures of cellular morphology. However, the differences in the sensitivities of features to segmentation were consistent with the methodology used to calculate those features. Features that were direct measures of cell body shape tended to be most different between well- and poorly-segmented populations, consistent with the presence of 4 cell-body shape-based features in our classifier. Nuclear staining quantitation was largely independent of the cell body channel, and was correspondingly insensitive to segmentation in the cell-body channel. Actin and tubulin features that were quantitated inside the region that was segmented in the cell-body channel had intermediate sensitivity to that segmentation. Finally, intensity and texture features tended to be less sensitive to segmentation than shape or dimension features, indicating that those features might be more robust than shape features in cases where cell-body segmentation is poor.

If a shape-based filter such as the one we describe is to be useful, it is critical that it leads to demonstrable improvements in data quality. To assess this we considered three aspects of our data in well- and poorly-segmented populations.

First, we assessed overall variation in our data, pre- and post- application of the shape filter, to determine if our filter reduced unwanted technical noise in the data, while retaining variation of biological interest. A filter that selected only (for example) G1-phase cells would reduce variation in the filtered morphology data, but at the cost of losing potentially valuable information from cells in other phases of the cell cycle. We found that our shape-based filter did not inappropriately reduce overall variation in most morphological features (Figure 5). This indicated that the well-segmented population of cells that passed our filter, retained variation of biological interest.

Second, we reviewed the DNA distributions of well- and poorly-segmented populations. This revealed that the poorly-segmented objects included a number of acellular debris objects with little or no detectable DNA content (Figure 4). These objects were largely eliminated by the segmentation filter, leading to a clear improvement in the quality of the cell population, by eliminating acellular debris. This was achieved without inappropriate specific loss of cells in any particular cell-cycle phase.

Finally, we queried the data for morphological features that had significant dose responses to specific drugs, and asked if the quantitative changes in those features in either well-segmented or poorly-segmented populations, captured real qualitative changes as assessed by visual inspection of the cells. In the cell body channel, we found that 17-AAG at high dose tended to make cells round up, consistent with a cell-cycle block around mitosis, or cell killing. This qualitative change was somewhat subtle (Figure 7a,b), but it was reflected in a significant decrease in perimeter-to-area ratio among well-segmented cells (Figure 6a). In contrast, this morphological change would not have been clearly detected if the poorly-segmented cells had been analyzed (Figure 6b). In the actin channel, a similar trend was seen with the actin fiber count. Among the well-segmented population, a significant decrease in actin fiber count occurred after treatment with high-dose Herbimycin (Figure 8a), as confirmed by visual inspection of images (Figure 9), but this trend was attenuated when only poorly-segmented cells were analyzed (Figure 8b).

## Conclusion

Commercial HCS systems are powerful tools for the elucidation of cellular morphology. One of the factors that can limit the quality of HCS data is imperfect segmentation of cells, especially in "HCS-unfriendly" cell lines. We have shown that a shape-based SVM-RBF classifier can reproduce a human classification of well- and poorly-segmented objects with 81% accuracy. Intensity and texture features tended to be more resistant to poor cell segmentation than shape or dimension features. Application of the shape-based classifier as a data filter yielded quantifiable improvements in data quality. DNA content measurements were cleared of a spurious debris signal, and discrimination of visually-evident morphological changes in cells was sharpened. These results highlight the importance of high-quality image segmentation in the analysis of HCS data.

## Methods

### Cell culture

SK-BR-3 cells were cultured at 37C, 5% CO_2 _in RPMI 1640 medium supplemented with 10% fetal bovine serum. 5000 cells in a total volume of 40 uL were plated per well of a 384 well black wall, clear bottom tissue culture plate (Becton Dickenson cat#35326).

### Compound treatment

A library of compounds was prepared in DMSO to a final concentration of 10 mM except for Herbimycin A (prepared at 1 mM). Compounds were diluted three fold serially down each column of a 384 well plate in DMSO. Four microliters from the DMSO compound plate were transferred to a dilution plate containing 60 uL media per well. An additional transfer of 8 uL from the dilution plate to the assay plate containing cells in 40 uL media was made for a total 96 fold dilution of compound. Cells were incubated with vehicle or compound for 24 hours.

### Fixation and staining for HCS

Prior to fixation, live cells were stained for 30 minutes by adding 12 uL of 5 X CMFDA (Molecular Probes cat# C7025) per well diluted in serum free media. Cells were then washed with pre-warmed PBS on a Biotek Elx405 plate washer programmed to leave behind 20 uL per well. 20 uL of 2X (8%) paraformaldehyde in PBS were added per well and fixation was allowed to proceed for 10 minutes. Cells were washed and permeabilized for 10 minutes with PBS/0.2% Triton-X100. Cells were stained for 1 hour with a 1:125 dilution of an anti beta-tubulin antibody (BD Pharmingen cat# 556321) diluted in PBS/1% Goat serum. Cells were then washed and stained for 1 hour with a cocktail of a 1:200 dilution of an Alexa labelled secondary antibody (Molecular Probes goat anti-mouse Alexa-647 cat# A21238), 4 U/mL Phalloidin Alexa-546 (Molecular Probes cat #A22283) for actin labelling, and 0.35 uM DAPI (Molecular Probes cat#D21490) diluted in PBS/1% goat serum, for DNA labelling. Following staining, cells were washed and plates were sealed for automated fluorescent microscopy.

### HCS image capture and processing

Cell images were captured in four channels using a 20X objective on a Cellomics ArrayScan VTi system (Cellomics, Pittsburgh, PA). The four channels corresponded to the cell body CMFDA stain (Ch1), the nuclear DAPI stain (Ch2), the actin stain (Ch3), and the tubulin stain (Ch4). Cells were segmented and feature values were computed from the 4-channel images by the Cellomics Morphology Explorer BioApplication version 5.0. A constant 20 fields per well were captured in autoexpose mode. Object segmentation in the Morphology Explorer BioApplication was done in each channel as described in the following paragraphs. Also refer to Chapter 2 of the Cellomics Morphology Explorer User Guide [12] for more details about specific segmentation parameters described below.

In Ch1 (cell body), isodata intensity thresholding was first applied. In this histogram-based method, the intensity threshold for objects is chosen by an iterative method so that it is equal to the average of the mean of the pixel intensities to the left of the threshold and the mean of the pixel intensities to the right of the threshold. The resulting threshold value was then multiplied by (1+IsoDataThreshold), where IsoDataThreshold was set to -0.999. No image smoothing was applied. After thresholding, watershed segmentation was applied to resolve overlapping objects, with Cellomics parameters ObjectSegmentationCh1 = 16, WatershedCh1 = 100.

In Ch2 (nucleus), isodata thresholding was applied in the same way as for channel 1, except that the IsoDataThreshold parameter was set to -0.35. No image smoothing was applied. To resolve nuclei, simple segmentation based on typical nuclear radius parameter (Cellomics terminology: MemberSegmentationCh2) of 6 pixels was done inside the region defined by the Ch1 object (Cellomics parameter ObjectMaskModifierCh2 = 0).

In Ch3 (actin) and Ch4 (tubulin), 3-sigma intensity thresholding was applied. In this method, the intensity threshold for objects was set to 3 times the standard deviation of the pixel intensities, multiplied by the factor (1+3SigmaThreshold), where 3SigmaThrehold was set to -0.988. No image smoothing was applied. After thresholding, spots/fibers were identified by spatial variation filtering with a half-width parameter (Cellomics terminology: SpotFiberSizeCh3 and SpotFiberSizeCh4) of 1 pixel. Objects were identified within the cytoplasmic region defined by Ch1 cell body object (Cellomics Parameters: ObjectMaskModifierCh3 = ObjectMaskModifierCh4 = 0)

A total of ~190 features were defined by the Morphology Explorer application, including measures of object position, orientation, intensity, size, shape, and texture. Of these, we focused our attention on two subsets of primary interest. First, for building classifiers, we considered the subset of 116 features that had defined values for every cell in our training set (feature data is available in additional file 1).

The calculation of morphological features is described in the Cellomics Morphology Explorer BioApplication Guide [12]. Here, we briefly describe nine features that were used in the SVM-RBF classifier, or shown in our results. Total intensity in Channels 2 and 3 was calculated for each object by integrating the total intensity in objects in these channels; average intensities in Channel 2 and 3 were the corresponding total intensities divided by the number of pixels in the object. Spot Fiber Count in Channel 3 was the count of identified actin fiber objects in each object. Convex hull to area ratio in Channel 1 was the ratio of the area of the convex hull of an object, to the area of the object. Convex hull perimeter ratio in Channel 1 was the ratio of the convex hull perimeter to the perimeter of the object. Finally, if *L *and *W *are defined as the length and width of the rectangle that bounds the cell body in Channel 1, then P2A and FW in Ch1 were defined as follows [12]:

P2A={cell perimeter}24π{cell area}
 MathType@MTEF@5@5@+=feaafiart1ev1aaatCvAUfKttLearuWrP9MDH5MBPbIqV92AaeXatLxBI9gBaebbnrfifHhDYfgasaacH8akY=wiFfYdH8Gipec8Eeeu0xXdbba9frFj0=OqFfea0dXdd9vqai=hGuQ8kuc9pgc9s8qqaq=dirpe0xb9q8qiLsFr0=vr0=vr0dc8meaabaqaciaacaGaaeqabaqabeGadaaakeaacqWGqbaucqaIYaGmcqWGbbqqcqGH9aqpdaWcaaqaaiabcUha7jabdogaJjabdwgaLjabdYgaSjabdYgaSjabbccaGiabdchaWjabdwgaLjabdkhaYjabdMgaPjabd2gaTjabdwgaLjabdsha0jabdwgaLjabdkhaYjabc2ha9naaCaaaleqabaGaeGOmaidaaaGcbaGaeGinaqdcciGae8hWdaNaei4EaSNaem4yamMaemyzauMaemiBaWMaemiBaWMaeeiiaaIaemyyaeMaemOCaiNaemyzauMaemyyaeMaeiyFa0haaaaa@58E2@

FW={cell perimeter}2−L
 MathType@MTEF@5@5@+=feaafiart1ev1aaatCvAUfKttLearuWrP9MDH5MBPbIqV92AaeXatLxBI9gBaebbnrfifHhDYfgasaacH8akY=wiFfYdH8Gipec8Eeeu0xXdbba9frFj0=OqFfea0dXdd9vqai=hGuQ8kuc9pgc9s8qqaq=dirpe0xb9q8qiLsFr0=vr0=vr0dc8meaabaqaciaacaGaaeqabaqabeGadaaakeaacqWGgbGrcqWGxbWvcqGH9aqpdaWcaaqaaiabcUha7jabdogaJjabdwgaLjabdYgaSjabdYgaSjabbccaGiabdchaWjabdwgaLjabdkhaYjabdMgaPjabd2gaTjabdwgaLjabdsha0jabdwgaLjabdkhaYjabc2ha9bqaaiabikdaYaaacqGHsislcqWGmbataaa@48A4@

Note that the definition of P2A is such that a perfectly circular object would have P2A = 1. From its definition, FW is also related to cell shape; as an object becomes more elongated its FW would approach 0.

### Definition of feature types

For the analysis of Table 3, we defined a subset of 57 of the 116 features used for building classifiers, by excluding "status" features. These status features are integer flag variables, which indicate that a specific morphological feature is inside or outside a user-defined range. For our analysis of feature types these status features were not relevant. We assigned a type to each of the 57 non-status features to indicate what facet of cell morphology each feature reflected: "intensity" for features that primarily reflected the intensity of staining, "shape" for dimensionless shape parameters, "texture" for intensity texture features, "arrangement" for features indicating object arrangement, and "dimension" for features related to object size.

### Calculation of Kolmogoroff-Smirnov statistics

As a statistical measure of changes in each morphological feature, we used the Kolmogoroff-Smirnoff (KS) statistic [13] to compare the distribution of each feature in a population of perturbed cells to the corresponding distribution in vehicle-treated cells on the same 96-well plate. This controlled for inter-plate level technical variation in the experimental process.

### Measurement of the sensitivity of features to segmentation

To assess the sensitivity of morphological features to segmentation, we fitted the dose-response of the KS statistic for each feature to each compound by a linear ANOVA model, using either well-segmented cells, or poorly-segmented cells. Then, the error-weighted discrepancy **D **between the fitted model from the well-segmented cell population and that from the poorly-segmented population was calculated as:

D=∑dosesabs(yws−yps)SEws
 MathType@MTEF@5@5@+=feaafiart1ev1aaatCvAUfKttLearuWrP9MDH5MBPbIqV92AaeXatLxBI9gBaebbnrfifHhDYfgasaacH8akY=wiFfYdH8Gipec8Eeeu0xXdbba9frFj0=OqFfea0dXdd9vqai=hGuQ8kuc9pgc9s8qqaq=dirpe0xb9q8qiLsFr0=vr0=vr0dc8meaabaqaciaacaGaaeqabaqabeGadaaakeaacqWGebarcqGH9aqpdaaeqbqaamaalaaabaGaemyyaeMaemOyaiMaem4CamNaeiikaGIaemyEaK3aaSbaaSqaaiabdEha3jabdohaZbqabaGccqGHsislcqWG5bqEdaWgaaWcbaGaemiCaaNaem4CamhabeaakiabcMcaPaqaaiabdofatjabdweafnaaBaaaleaacqWG3bWDcqWGZbWCaeqaaaaaaeaacqWGKbazcqWGVbWBcqWGZbWCcqWGLbqzcqWGZbWCaeqaniabggHiLdaaaa@4CEE@

where y_ws _was the fitted estimate from well-segmented cells, y_ps _was the estimate from poorly-segmented cells, and SE_ws _was the residual standard error of the ANOVA model for the well-segmented cells. Hence D was a measure of how different the dose responses for each drug-feature combination were, if estimated from either well-segmented cells alone, or poorly-segmented cells alone. For the analysis of Table 3, drug-feature combinations with D less than the median D of all drug-feature combinations were called "resistant", and drug-feature combinations with D greater than the median D were called "sensitive".

### Data analysis software

Automated segmentation of images was carried out by the Cellomics HCS View application (Cellomics, Pittsburgh, PA). Images were reviewed and manipulated using MATLAB (version R2006a, MathWorks, Natick, MA). Statistical analysis of feature data was executed in the R statistics package (version 2.1.1, http://www.r-project.org).

#### Competing interests

The author(s) declares that there are no competing interests.

## Authors' contributions

PL did all cell culture and image acquisition. AH, SH, PL, and YL all manually curated cells for the training and test sets. AH executed the classification analysis. YL developed quality assessment and feature analysis methods that were used on the data. SH initiated and directed the project. All authors read and approved the final manuscript.

## Supplementary Material

Additional file 1 Feature data for 2019 training and test cells. The structure of the data table is as follows. The first column contains unique cell identifiers. The first row contains headers, including: "Case" – indicates if the cell was randomly assigned to the training or test sets "Class" – the true class of the cell by human review (poorly segmented (PS) or well-segmented (WS) ) The remaining 116 columns contain the feature data for each cell. A total of ~190 features were generated by the Cellomics Morphology Explorer software, but only a subset of 116 features with complete data for every cell were included in our analysis. Click here for file
